# Study Protocol of a Prospective Phase 2 Study of Chlorophyllin for the Management of Brain Radionecrosis in Patients With Diffuse Glioma (CHROME)

**DOI:** 10.1002/cam4.70657

**Published:** 2025-03-02

**Authors:** Archya Dasgupta, Saranga Sawant, Abhishek Chatterjee, Vikram Gota, Arpita Sahu, Amitkumar Choudhari, Kajari Bhattacharya, Ameya Puranik, Indraja Dev, Aliasgar Moiyadi, Prakash Shetty, Vikas Singh, Nandini Menon, Sridhar Epari, Ayushi Sahay, Aekta Shah, Nazia Bano, Farnaz Shaikh, Aabha Jirage, Tejpal Gupta

**Affiliations:** ^1^ Department of Radiation Oncology Tata Memorial Centre Mumbai India; ^2^ Homi Bhabha National Institute (HBNI) Mumbai India; ^3^ Clinical Research Secretariat (CRS), Tata Memorial Centre Mumbai India; ^4^ Department of Clinical Pharmacology The Advanced Centre for Treatment, Research and Education in Cancer, Tata Memorial Centre Navi Mumbai India; ^5^ Department of Radio‐Diagnosis Tata Memorial Centre Mumbai India; ^6^ Department of Nuclear Medicine Tata Memorial Centre Mumbai India; ^7^ Neurosurgical Oncology Services, Department of Surgical Oncology Tata Memorial Centre Mumbai India; ^8^ Department of Medical Oncology Tata Memorial Centre Mumbai India; ^9^ Department of Pathology Tata Memorial Centre Mumbai India

**Keywords:** chlorophyllin, diffuse glioma, glioblastoma, high‐grade glioma, radionecrosis

## Abstract

**Introduction:**

Chlorophyllin (CHL) effectively decreases the side effects of radiotherapy (RT) by scavenging radiation‐induced free radicals and reactive oxygen species in preclinical trials. This study aims to assess the efficacy of oral CHL for the treatment of brain radionecrosis in patients with diffuse glioma.

**Methods:**

This is a phase 2 trial prospective, interventional study. Adults (> 18 years) with a histological diagnosis of diffuse glioma developing radionecrosis will be eligible for the study. Radionecrosis will be identified using standard imaging protocols with magnetic resonance imaging (MRI) with or without positron emission tomography (PET). Patients will be accrued in two strata: symptomatic (stratum A) and asymptomatic (stratum B). Chlorophyllin will be prescribed to all patients using a morning oral dose of 750 mg before breakfast for 3 months. In addition, participants in stratum A will be given a tapering dose of dexamethasone for 1 month, while stratum B will not be receiving any steroids. Imaging with an MRI brain protocol and PET scan will be planned at 1 month and MRI at 3 months after starting CHL. The primary endpoint is the clinical‐radiological response at 1 month. Secondary endpoints include response at 3 months, biological responses, survival analysis, and quality‐of‐life scores. The total sample size is 118 (60 and 58 in stratum A and B, respectively), with one interim analysis planned.

**Discussion:**

Radionecrosis leads to significant morbidity and is usually treated with corticosteroids, which can lead to several side effects from both acute and long‐term use. Refractory radionecrosis requires treatment with bevacizumab or surgical resection. Chlorophyllin is a cheap, safe, and readily available phytopharmaceutical drug, which is being investigated in the phase 2 study and, if proven effective, can be considered an alternative for treating radionecrosis.

**Trial Registration:** Clinical Trial Registry India (CTRI): CTRI/2023/08/056166; ClinicalTrials.gov: NCT06016452

## Graphical abstract

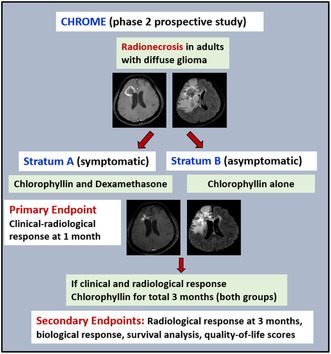

**Abbreviations**
CHL: chlorophyllin
CT: computed tomography
CTCAE: common terminology criteria for adverse events
CTRT: chemotherapy radiotherapy
EORTC: European Organization for Research and Treatment of Cancer
GCP: good clinical practice
ICD: informed consent document
ICF: informed consent form
KPS: Karnofsky performance scale
MRI: magnetic resonance imaging
NANO: neurologic assessment in neuro‐oncology
NPS: neurological performance scale
PET: positron emission tomography
PFS: progression free survival
QOL: quality of life
QTWiST: quality‐adjusted time without toxicity
RN: radionecrosis
RT: radiotherapy
WBI: whole brain irradiation

## 1 Introduction

Radiotherapy (RT) plays an integral role in the multimodality management of adult‐type diffuse gliomas. Radiation is indicated in adult‐type low‐grade gliomas with high‐risk features or high‐grade gliomas following maximal safe resection [1, 2, 3]. Higher doses of RT can lead to symptomatic radio‐necrosis (RN) in approximately 5%–15% of patients, typically within the first 2 years of RT completion [4, 5, 6, 7]. The development of RN can lead to significant morbidity, with new‐onset or worsening of pre‐existing neurodeficits having substantial implications for quality of life (QOL), and in extreme situations, can lead to mortality. The pathogenesis of RN is multifactorial, with a complex interplay of vascular‐mediated damage and injury to glial cells primarily postulated through the activation of several inflammatory markers like tumor necrosis factor, interleukins, and vascular endothelial growth factor [6]. Corticosteroids, preferably dexamethasone, form the first line of management of RN, with variable response rates ranging from 25%–60%, impacted by several factors like the dose of RT and response evaluation methods (neurological/radiographic) [8, 9]. The response rate in our practice concurs with the reported literature, with combined clinical and radiological responses seen in approximately 50% of patients from institutional experience and audit. It is important to note that the long‐standing use of corticosteroids comes at the cost of complications like hyperglycemia, myopathy, and increased risk of infections precluding prolonged use. Also, a proportion of patients remain refractory to steroids or turn out to be dependent on steroids, where bevacizumab (anti‐angiogenic agent) can be used as second‐line therapy in appropriately selected patients [10, 11]. However, the major disadvantages of bevacizumab remain intravenous administration, requiring regular hospital visits, treatment costs, and concerns for related toxicities like hypertension and intracranial or extracranial hemorrhage. Other agents like hyperbaric oxygen therapy, pentoxifylline, and tocopherol have been suggested in refractory radionecrosis, with questionable benefits [6].

Sodium‐copper‐chlorophyllin is a phytopharmaceutical drug obtained from the green plant pigment chlorophyll. It is a semi‐synthetic mixture of sodium copper salts derived from chlorophyll. Chlorophyllin scavenges RT‐induced free radicals and reactive oxygen species. It has been used as a food colorant and over the counter in the USA, Japan, Australia, and China for many years for various health benefits, including the prevention of body odor in geriatric patients, enhanced wound healing, antibacterial action, etc. [12]. Studies have shown that CHL has immunostimulatory, anti‐inflammatory, and antiviral effects in addition to antioxidant and radioprotective properties [13]. It increases the expression of a transcription factor (protein) Nrf2, improving lymphocyte survival and enabling efficient detoxification after RT exposure [14, 15].

The current study is a phase 2 study to investigate the role of CHL as an anti‐inflammatory agent in treating brain radionecrosis, a long‐term toxicity of cranial radiotherapy.

## 2 Study Methodology

### 2.1 A. Study Design/Population

**FIGURE 1:**
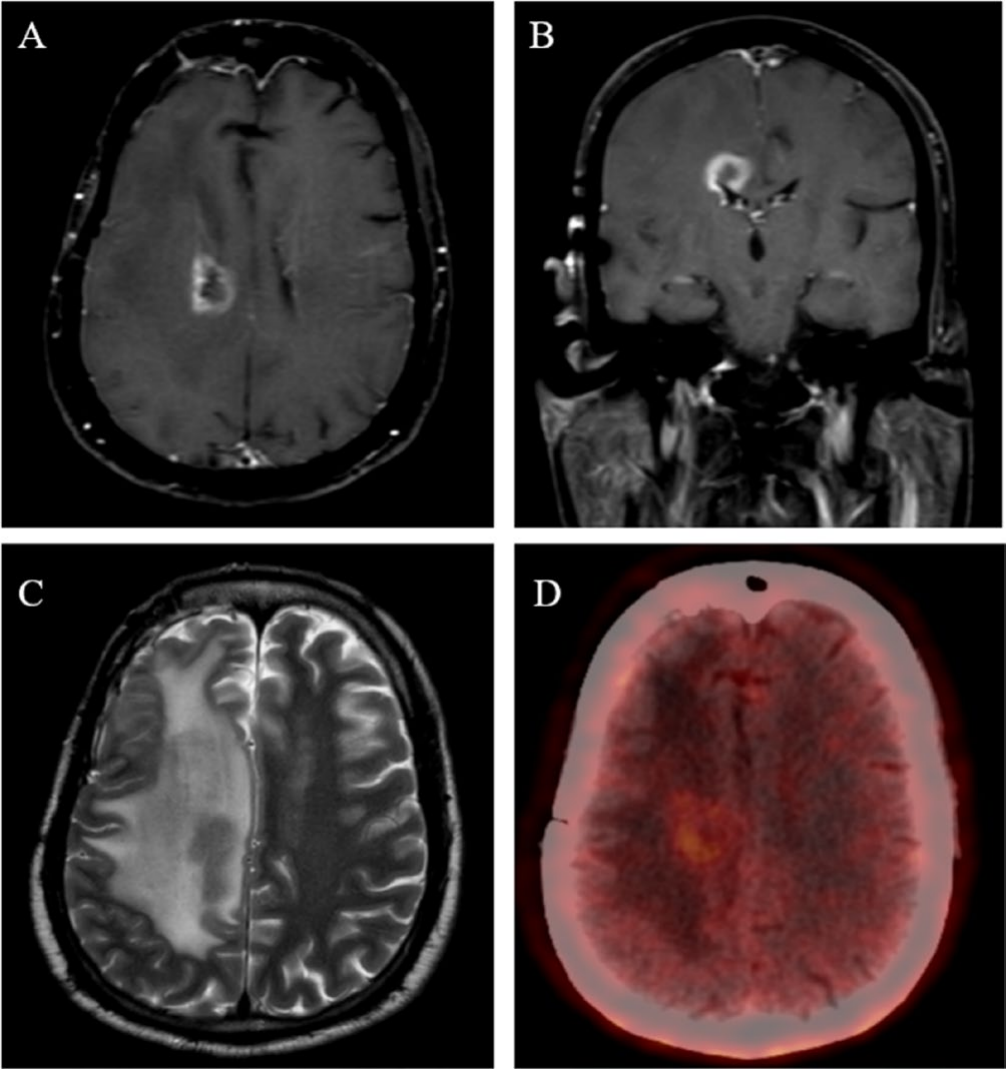
Radiological findings for a patient with radionecrosis. (A, B) show the presence of an enhancing rim with a central necrotic core along the right periventricular region. (C) is the corresponding T2‐weighted image showing significant edema. (D) is the F‐DOPA PET showing low‐grade avidity of the lesion.

This is a prospective phase 2 open‐label study conducted in a single institute. Patients with a histopathological diagnosis of adult‐type diffuse glioma treated with conventionally fractionated high‐dose radiotherapy developing radionecrosis will be eligible for the current study. Radionecrosis will be diagnosed using standard institutional protocols based on magnetic resonance imaging (MRI) and/or positron emission tomography (PET) as decided clinically. MRI will include standard brain protocol per institutional practice, including T1w pre‐ and post‐contrast, T2w propeller, T2 FLAIR, GRE/susceptibility sequences, ADC, DWI, spectroscopy, and perfusion‐weighted imaging. All the images will be reviewed independently by a neuroradiologist and nuclear medicine physician (as appropriate) and discussed in the multidisciplinary tumor board (in cases of indeterminate findings) to reach a consensus of RN and exclude the possibility of disease progression. For evaluating disease progression, the updated Response Assessment in Neuro‐Oncology criteria (RANO 2.0) will be used [16]. A patient with typical radiological findings of radionecrosis is presented in Figure 1. Patients will be assessed for the eligibility criteria below prior to inclusion in the study.

### 2.2 Inclusion Criteria

1. The histological diagnosis should be of adult‐type diffuse glioma.
2. Radionecrosis on imaging with new neurological symptoms or worsening of prior deficits (Stratum A) or without new symptoms (Stratum B).
3. The patient should be ≥ 18–70 years old during study accrual.
4. The Karnofsky Performance Scale (KPS) should be ≥ 50.

### 2.3 Exclusion Criteria

1. No tissue diagnosis.
2. Patients with KPS < 50.
3. Disease progression cases must be excluded.
4. Contraindications to corticosteroids.
5. Altered mental status with deficits in understanding or an inability to consent to the study.
6. Brainstem glioma diagnosis patients should be excluded
7. Indeterminate for radionecrosis vs. disease progression
8. The patient undergoing prior treatment with bevacizumab (either for disease progression or radionecrosis)

### 2.4 Study Intervention

After meeting study eligibility, written consent forms will be obtained from all the patients. Patients with symptomatic RN (new‐onset neurodeficit or worsening of prior deficit concurrently with imaging evidence of RN) and asymptomatic RN (no deficits or stable neurological status during imaging development of RN) will be accrued in strata A and B, respectively. The clinical status will be documented using the Neurological Performance Status (NPS) scale [17] and the Neurologic Assessment in Neuro‐Oncology (NANO) scale [18]. Patients in strata A will be treated with a tapering schedule of dexamethasone as per institutional practice, starting with 8 mg TDS for 5 days, followed by 4 mg TDS for 5 days, 4 mg BD for 5 days, 2 mg BD for 5 days, 1 mg BD for 5 days, and 1 mg OD for 5 days (cumulative duration 1 month). Patients in strata B will not be prescribed any steroids. Chlorophyllin will be prescribed to all patients using a morning oral dose of 750 mg before breakfast for a planned duration of 3 months. Imaging with an MRI brain protocol and amino acid PET scan will be scheduled at 1 month (additional examinations per study protocol) and MRI at 3 months (standard of care imaging) after starting CHL therapy. Repeat clinical evaluations with KPS, NPS, NANO, and QOL assessments will be done for all the patients at 1 month and 3 months from starting CHL.

### 2.5 Response Evaluation

The study endpoints have been summarized in Table 1. Response to CHL will be considered as the absence of all three criteria as follows:

1. Imaging progression of radionecrosis: Defined as an increase in T1‐contrast or T2w hyperintensity of the index lesion on the MRI brain, as assessed by a neuroradiologist. The PET scan at 1 month will be assessed by the nuclear medicine physician, and findings will be interpreted based on the avidity of the index lesion.
2. Clinical deterioration will be considered as a ≥ 2‐point drop in the NPS at assessment points (1 and 3 months) from baseline NPS.
3. Dexamethasone requirement: Any increase in the dexamethasone requirement during the predetermined tapering schedule will be considered refractory RN for stratum A. Any requirement of dexamethasone after CHL will be considered a failure of CHL in stratum B. Similarly, after the 1st month (when no dexamethasone use is planned in stratum A), the use of dexamethasone will be considered a failure of CHL for stratum A during the analysis of response at the 3rd month.

**TABLE 1:** Objectives and endpoints of CHROME study.

Study objective	To assess the efficacy of oral CHL in the treatment of radionecrosis in patients with diffuse glioma
Study endpoints	
Primary endpoint	Response rates (clinical‐radiological) at 1 month with the use of CHL
Secondary endpoint	Clinical‐radiological response rates at 3 months Biological response rates using functional imaging (PET and MRI) Survival analysis (Progression‐free survival and overall survival) EORTC QOL C‐30 and BN‐20 questionnaire at baseline, 1‐month, and at 3‐month follow‐ups NANO scale score QTWIST score
Exploratory endpoint	Temporal changes of inflammatory and tumor‐related biomarkers obtained from the serum

**FIGURE 2:**
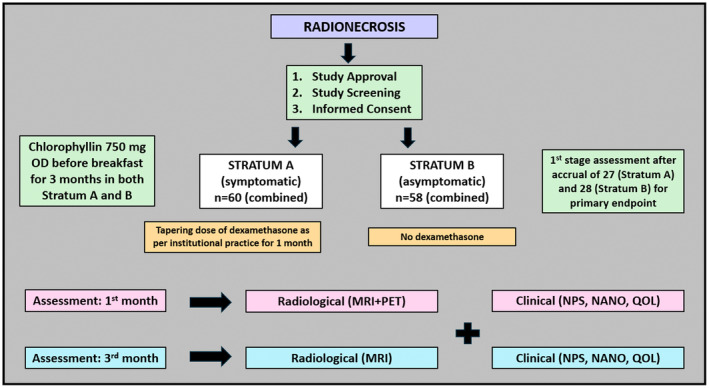
Schematic representation of the conduct of the study.

Therefore, for study analysis, combined evaluation (radiological, clinical, steroid requirement) will serve as a method of testing the efficacy of CHL, with progression in any single domain of the above three considered a failure, as mentioned above. Patients with unequivocal disease progression on imaging at 1 or 3 months will be excluded from RN response assessment (since the possibility of recurrence component is likely to contribute to neurological functioning and outcomes). Any discrepancies or equivocal findings will be discussed in a joint neuro‐oncology meeting (JNOM) for a consensus decision. No additional follow‐up visits will be required as per study protocol since patients with RN are called at 1 month for clinical evaluation and at 3 months for radiological and clinical evaluation. However, additional imaging will be undertaken with MRI and PET scan at 1 month after starting CHL. Imaging with MRI brain tumor protocol will be done at 3 months as the standard of care following institutional practice. The study workflow has been presented in Figure 2. A subgroup analysis of response to chlorophyllin will be done for patients with IDH‐mutant and IDH‐wild gliomas for both strata. As a part of the study (exploratory endpoint), 12–15 mL blood samples (EDTA and plain tubes) will be collected at baseline, 1 month, and 3 months. The blood will be analyzed for inflammatory indices from routine hemogram serum biochemistry, including albumin, CRP, and ferritin, along with additional exploratory markers like cytokines, chemokines (GM‐CSF, IFN‐gamma, TNF‐beta, interleukins, etc.), oxidative stress markers, proteomics, and metabolomics.

### 2.6 Sample Size Calculation

The proposed phase II prospective study will be split into two strata: Stratum A (symptomatic RN) and Stratum B (asymptomatic RN). Simon's two‐stage design (minimax) was used to calculate the sample size for each stratum, which is presented in Table 2.

**TABLE 2:** Sample size of the study with power estimates.

Stratum	Standard response rates	Expected response rates	Type 1 error	Power	No. of accrual 1st stage	No. of responses expected at 1st stage	Evaluation sample size at 1st stage (accounting for attrition 20%)	Total number of accrual needed for final analysis	Total no. of response expected for final analysis	Total sample size (considering attrition 20%)
Stratum A (Symptomatic)	50%	65%	0.0977	0.8022	22	10	27	50	29	60
Stratum B (Asymptomatic)	30%	45%	0.0963	0.8027	23	6	28	48	18	58

### 2.7 Stratum A

The response rate at 1 month with a combination of steroids and CHL in this study is expected to be 65% as compared to the standard 50% response with the use of dexamethasone alone (based on institutional experience and available literature) [7, 8, 9, 19]. For the study with a α of 0.1 and power of 80%, 50 patients will be needed to achieve the desired output. In the first stage, 22 patients will be needed for assessment and continue to stage 2 if > 10 responses are seen. Stage 2 of the study will be considered successful if > 29 responses are achieved using the pre‐specified response assessment criteria. Further considering an attrition rate of 10% from lack of follow‐up and another 10% for disease progression, an estimated 60 patients will be accrued in stratum A with the purpose of achieving 50 patients with endpoints available for analysis.

### 2.8 Stratum B

This stratum includes patients without neurological worsening during the concerned imaging diagnosis of RN (asymptomatic RN). Based on the available literature and institutional experience, in this group, approximately 30% of patients continue to be neurologically/radiologically stable or show regression of imaging findings without the need for further interventions (including corticosteroids) in the short term [19, 20, 21]. In the proposed study with the use of CHL, 45% of patients are assumed to remain clinically and neurologically stable. With a α of 0.1 and a power of 80%, 48 patients will be needed to achieve the desired outcome. In the first stage, 23 patients will be needed for assessment and will continue to stage 2 if > 6 responses are seen. The phase 2 study will be considered successful if > 18 responses are achieved using the pre‐specified response assessment criteria. Further considering an attrition rate of 10% from lack of follow‐up and another 10% for disease progression, an estimated 58 patients will be accrued in stratum B with the purpose of achieving 48 patients with endpoints available for analysis.

### 2.9 Statistical Analysis

Response rates will be analyzed using descriptive statistics. The influence of patient, disease, and treatment‐related factors on the response (binary outcomes) will be analyzed using Pearson Chi‐square or Fisher's exact test as appropriate. Survival analysis will be done using the Kaplan–Meier product‐limit method, considering the date of study accrual as the baseline. Univariate and multivariate analyses will be done using the log‐rank test and Cox regression, respectively. For exploratory analysis, the biomarkers will be compared using the t‐test and ANOVA, as appropriate. A *p*‐value of < 0.05 will be considered statistically significant. All data collection and analysis will be done using International Business Machines, Statistical Package for the Social Sciences (IBM SPSS) version 29.

## 3 Discussion

Brain radionecrosis is encountered in patients receiving high‐dose cranial radiotherapy, either with fractionated radiotherapy or ablative high‐dose radiosurgical treatments. Symptomatically, RN can present with an entire spectrum from radiologically evident clinically silent asymptomatic to life‐threatening situations refractory to treatment [22]. There are limited pharmacological options for effective treatment of RN other than corticosteroids and bevacizumab, which have their limitations regarding side effects. The current study will address the gap in investigating the role of chlorophyllin, a phytopharmaceutical with potential anti‐inflammatory effects in treating brain radionecrosis.

Radionecrosis is mostly seen within the first 2 years, with the most common time being 6–18 months post‐radiation. Factors influencing the incidence and severity of RN include radiation dose (with higher rates > 60 Gy EQD2), radiotherapy technique, radiation volume, fractionation, and other biological factors, including intrinsic radiosensitivity [22, 23]. The diagnosis of radionecrosis can often be challenging due to the similar radiological appearance with disease recurrence, which should be ruled out as prognosis and treatment implications are completely different. The use of multiparametric imaging, including advanced functional sequences like perfusion‐weighted imaging and MR spectroscopy, can help differentiate radionecrosis from recurrence [24]. Typically, the changes appearing within the initial few months from radiation in the high‐dose region, the presence of Swiss cheese appearance on the T1‐contrast sequence, and hypointense to hypointense signal of T2‐weighted imaging should lead to suspicion for radionecrosis. Compared to recurrence, radionecrosis is typically hypoperfused on perfusion imaging and has a lower Choline: NAA peak on MR spectroscopy. Dynamic susceptibility contrast MRI has proven to be useful in differentiating radionecrosis from tumor recurrence, and standardized protocols have been recommended by consensus [25, 26]. Novel imaging techniques like treatment response assessment maps (TRAMs) which utilize multiple T1‐contrast sequences to explore differential washout of contrast from viable tumor and necrotic regions, or T1 mapping techniques can provide further clarity in differentiating radiation‐induced changes from disease recurrence [27, 28]. Amino acid PET with carbon‐11 methionine, DOPA, and FET can add to the MRI findings in improving the diagnostic performance in differentiating between radionecrosis and disease progression, with sensitivity and specificity of 80%–95%, as shown in different studies [29, 30, 31, 32, 33, 34]. In the current study, the patients will undergo multiparametric MRI, including spectroscopy, perfusion, and diffusion‐weighted imaging, to diagnose radionecrosis. In equivocal cases, additional imaging with FET or F‐DOPA will be considered for confirmation of the diagnosis. All the patients will be discussed in the multidisciplinary joint neuro‐oncology meeting before accrual in the study. The interval imaging after starting intervention (starting CHL) will be considered at 1 month with both MRI and PET for assessment of both morphological and functional response for primary endpoint assessment. Further imaging will be considered at 3 months for evaluation of long‐term response to CHL. Since there is always a possibility of inclusion of patients with progression, these patients will be excluded from analysis regarding the efficacy of CHL if recurrence is documented within 3 months from study accrual.

The most commonly used medication for RN is corticosteroids, which can relieve symptoms and lead to reversal or stability of radiological findings in a proportion of patients. There are no guidelines regarding the dose and duration of use of corticosteroids and also the type of steroids (with dexamethasone preferred by the majority). Typically, in symptomatic patients, at least a few weeks of steroid treatment are required for optimal clinical benefit. There are concerns about certain adverse effects both with short‐and long‐term use of steroids, including insomnia, acute psychosis, hyperglycemia, osteoporosis, risk of infections, skin changes, myopathy, and adrenocortical insufficiency, which can itself lead to new complications affecting quality of life. Also, some patients often turn into a state of steroid dependence with relapse of symptoms upon withdrawal of steroids. In refractory cases, the anti‐angiogenic agent bevacizumab can provide significant benefit; however, the major challenges remain the need for frequent hospital visits for intravenous administration (typically delivered every 2–3 weeks), associated costs, and side effects including hypertension or life‐threatening hemorrhages. Some agents with questionable benefits include edaravone, pentoxifylline, and hyperbaric oxygen therapy [8, 35]. The current study will use CHL to treat radionecrosis, which has demonstrated anti‐inflammatory and antioxidant properties with a free radical scavenging effect in preclinical studies, with the pathophysiology of radionecrosis being considered a pro‐inflammatory environment [15, 36]. In the study, we are considering patients with symptomatic and asymptomatic radionecrosis in two different strata since the burden of necrosis, tempo of disease, and clinical outcomes are expected to be different. In the symptomatic stratum, patients will be treated with CHL in addition to oral dexamethasone, which is otherwise used as the standard first‐line therapy for symptomatic RN in our institution, while in the other stratum (asymptomatic), patients will receive CHL alone to explore the role of CHL as a steroid‐sparing agent.

The primary endpoint used in the study includes combined clinical and radiological criteria at 1 month for assessment of response. Other endpoints include 3‐month response and additional patient‐reported outcomes, survival outcomes, toxicity, and exploratory analysis for blood markers, which can predict the response to treatment. If proven useful and the primary endpoint is met, further phase 3 randomized studies will be planned to determine the efficacy of CHL in the treatment of radionecrosis. If proven useful, this will open a new avenue for the treatment of radionecrosis as a well‐tolerated oral therapy, avoiding or reducing the side effects of current standard treatment with corticosteroids.

## 4 Conclusion

Chlorophyllin is an orally administered phytopharmaceutical that has anti‐inflammatory effects. In the current phase 2 study, the efficacy of CHL will be investigated in both symptomatic and asymptomatic radionecrosis, with clinico‐radiological response at 1 month as the primary endpoint.

## Author Contributions

**Archya Dasgupta:** conceptualization (lead), data curation (lead), formal analysis (lead), funding acquisition (lead), investigation (lead), methodology (lead), project administration (lead), resources (lead), writing – original draft (lead). **Saranga Sawant:** data curation (equal), investigation (equal), methodology (equal), writing – original draft (equal). **Abhishek Chatterjee:** conceptualization (equal), data curation (equal), formal analysis (equal), investigation (equal), methodology (equal), project administration (equal), writing – review and editing (equal). **Vikram Gota:** data curation (equal), funding acquisition (lead), investigation (equal), methodology (equal), writing – review and editing (equal). **Arpita Sahu:** data curation (equal), investigation (equal), methodology (equal), writing – review and editing (equal). **Amitkumar Choudhari:** data curation (equal), investigation (equal), methodology (equal), writing – review and editing (equal). **Kajari Bhattacharya:** data curation (equal), investigation (equal), methodology (equal), writing – review and editing (equal). **Ameya Puranik:** data curation (equal), investigation (equal), methodology (equal), writing – review and editing (equal). **Indraja Dev:** data curation (equal), investigation (equal), methodology (equal), writing – review and editing (equal). **Aliasgar Moiyadi:** data curation (equal), investigation (equal), methodology (equal), writing – review and editing (equal). **Prakash Shetty:** data curation (equal), investigation (equal), methodology (equal), writing – review and editing (equal). **Vikas Singh:** data curation (equal), investigation (equal), methodology (equal), writing – review and editing (equal). **Nandini Menon:** data curation (equal), investigation (equal), methodology (equal), writing – review and editing (equal). **Sridhar Epari:** data curation (equal), investigation (equal), methodology (equal), writing – review and editing (equal). **Ayushi Sahay:** data curation (equal), investigation (equal), methodology (equal), writing – review and editing (equal). **Aekta Shah:** data curation (equal), investigation (equal), methodology (equal), writing – review and editing (equal). **Nazia Bano:** data curation (equal), investigation (equal), methodology (equal), writing – review and editing (equal). **Farnaz Shaikh:** data curation (equal), investigation (equal), methodology (equal), writing – review and editing (equal). **Aabha Jirage:** data curation (equal), investigation (equal), methodology (equal), writing – review and editing (equal). **Tejpal Gupta:** conceptualization (equal), data curation (equal), formal analysis (equal), investigation (equal), methodology (equal), project administration (equal), writing – review and editing (equal).

## Ethics Statement

The study is being conducted in accordance with ICMR (2017) “National Ethical Guidelines for Biomedical and Health Research Involving Human Participants, International Conference on Harmonization Good Clinical Practice (ICH‐GCP)” guidelines, Good Clinical Practice, and the principles of the Declaration of Helsinki. The study, including all the study‐related documents, has obtained approval from the Ethics Committee prior to the enrollment of participants. The study is registered with the Clinical Trial Registry, India (CTRI) and ClinicalTrial.gov.

## Consent

The authors have nothing to report.

## Conflicts of Interest

The authors declare no conflicts of interest.

## Data Availability

Data will be provided upon reasonable request to the principal investigator following the guidelines of the institutional ethics committee.
